# Patient safety incident capture resulting from incident reports: a comparative observational analysis

**DOI:** 10.1186/s12873-015-0032-7

**Published:** 2015-04-11

**Authors:** Martin A Reznek, Kevin A Kotkowski, Michael W Arce, Zachary K Jepson, Steven B Bird, Chad E Darling

**Affiliations:** Department of Emergency Medicine, University of Massachusetts Medical School, 55 Lake Avenue North, Worcester, MA 01655 USA

**Keywords:** Incident reporting, Quality improvement, Patient safety, Emergency medicine

## Abstract

**Background:**

Patient safety incident (PSI) discovery is an essential component of quality improvement. When submitted, incident reports may provide valuable opportunities for PSI discovery. However, little objective information is available to date to quantify or demonstrate this value. The objective of this investigation was to assess how often Emergency Department (ED) incident reports submitted by different sources led to the discovery of PSIs.

**Methods:**

A standardized peer review process was implemented to evaluate all incident reports submitted to the ED. Findings of the peer review analysis were recorded prospectively in a quality improvement database. A retrospective analysis of the quality improvement database was performed to calculate the PSI capture rates for incident reports submitted by different source groups.

**Results:**

363 incident reports were analyzed over a period of 18 months; 211 were submitted by healthcare providers (HCPs) and 126 by non-HCPs. PSIs were identified in 108 resulting in an overall capture rate of 31%. HCP-generated reports resulted in a 44% capture rate compared to 10% for non-HCPs (*p* < 0.001). There was no difference in PSI capture between sub-groups of HCPs and non-HCPs.

**Conclusion:**

HCP-generated ED incident reports were much more likely to capture PSIs than reports submitted by non-HCPs. However, HCP reports still led to PSI discovery less than half the time. Further research is warranted to develop effective strategies to improve the utility of incident reports from both HCPs and non-HCPs.

## Background

In 1999, the Institute of Medicine presented startling findings that 44,000 to 98,000 patients die annually due to medical errors [1]. Since that report, patient safety has become a major priority in healthcare.

Incident reporting is important for improving patient safety because it can lead to the identification of patient safety incidents (PSIs), defined by the World Health Organization as “event[s] or circumstance[s] that could have resulted, or did result, in unnecessary harm to a patient” [2]. PSI capture is a crucial step in patient safety improvement because it enables subsequent development and implementation of preventative measures [3]. In Emergency Medicine (EM), potential incidents are reported to Emergency Department (ED) leadership from a wide variety of sources including ED healthcare providers (HCPs), HCPs in other departments, hospital quality officers, hospital committees, as well as patients and their families. These reports may represent valuable opportunities for discovery of true PSIs, however little objective information is available to quantify or demonstrate this value. In EM, a limited number of studies suggest that patient complaints may be useful in improving quality [4,5], and one study has implied that physician-generated concerns may be more valuable for quality improvement [6]. To our knowledge however, there are no studies in any field of healthcare that have specifically evaluated the efficacy of incident reports from different sources in identifying PSIs.

The objective of this investigation was to assess how often ED incident reports from different sources led to the identification of PSIs.

## Methods

### Study design and setting

Data for this observational study were collected between October 2010 and April 2012 at an urban, tertiary care ED with an approximate annual census of 66,000 adult patients. The ED is the primary teaching site for an accredited 3-year EM residency. Board-certified EM physicians care directly for approximately 95% of the patients either as the only provider or through direct supervision of EM and rotating residents. Mid-level practitioners care for approximately 5% of the patients as the primary provider. The University of Massachusetts Medical School Institutional Review Board reviewed and approved the study.

### Methods and measurements

To analyze incident reports submitted to the ED, a standardized peer review process was implemented in October 2010. The process was based on one previously described at Detroit Receiving Hospital (DRH) [7], however we modified the DRH process also to include systems failure identification and analysis. As a matter of policy, every incident report submitted to the ED was analyzed via this process which is described below.

As a first step, each incident report underwent a preliminary screening review. Due to practical considerations, we opted for a single-reviewer format for the preliminary screening. To minimize potential accuracy and bias limitations inherent to a single-reviewer screening, the chosen preliminary reviewer had greater than ten years of experience in healthcare quality and the incident reports could only be closed if it could be concluded with absolute certainty that no PSI had occurred. If a PSI could not be excluded with absolute certainty, the incident report was forwarded to the ED Peer Review Committee (PRC) for analysis.

Convening monthly, the PRC was open to all ED physicians, residents and mid-level practitioners, however eight board-certified, attending physicians attended regularly to provide consistency in the evaluation process. Prior to each PRC meeting, ED practitioners involved with a case to be reviewed were required to provide a written account of the care and any other circumstances related to the specific quality issue(s) in question. This allowed for first-hand accounts of the care of the patient that might not be readily available in the medical record to be available to the PRC. In addition, the involved providers were encouraged to attend the PRC meeting. The medical records (and the practitioners’ written feedback if they were not present) were de-identified to foster a non-punitive and objective environment and to maintain patient confidentiality.

Facilitated by the PRC Chair, the committee reviewed each case for potential PSIs which were further subdivided by the PRC into six types of systems failures or contributing factors and five types of practitioner-based errors (Table 1). The classification scheme was developed based on schemes previously reported in EM literature. The classification framework of practitioner-based errors was modeled on one reported by Berk et al. [7]. For systems failures, the framework was developed based on portions of one suggested by Cosby [8]. Within the PRC review process, determination of a failure or error occurred regardless of correlation or causation of patient harm, and therefore near-misses were included as PSIs. A majority vote determined the presence or absence of systems failures and/or practitioner-based errors. (Providers involved in a case were required to leave the room during the vote on practitioner-based error for that case in order to reduce possible influence on the objectivity of the process.) To clarify the PSI identification and classification process, the following scenario is used as an example. A patient is admitted from the ED to an in-patient unit, and the patient’s condition subsequently deteriorates shortly after admission. The admitting team submits a concern that the care in the ED may have been suboptimal. Upon investigation, the ED peer review committee may determine that a systems failure did occur such as the patient did not receive an ordered medication due to a communication error. Or it may be determined after review, that the care was optimal, no systems failures or practitioner-based errors occurred, and the patient had simply deteriorated due to the natural course of disease. In the former outcome, a PSI was determined to have occurred, and in the latter outcome, it was determined that there was no PSI.

**Table 1:** **Patient safety incident types identified by the peer review committee**

**PSI type**	**Definition**	**Example**
**Systems failures**		
Triage	A failure in assessment of potential disease severity during triage	Abnormal vital signs not recognized as a potential sign of shock
ED teamwork	A failure due to an issue with ED staff communication or a shared responsibility across multiple ED staff	Change in vital signs not communicated to the attending physician
Hospital Teamwork	A failure due to an issue with communication between ED and hospital staff or a shared responsibility between the ED and hospital staff	Pertinent information not communicated to the admitting team
ED work environment	A failure resulting from the lack, malfunction, or mal-design of resources, equipment or physical space within the ED or a failure resulting from following an established ED policy or clinical practice guideline	Missing equipment needed for care of a patient
Hospital work environment	A failure resulting from the lack, malfunction, or mal-design of resources equipment or physical plant outside the ED but still within the hospital or a failure resulting from following an established hospital policy or guideline	Specialty testing areas remotely located from the ED causing prolonged transport time
Boarded patient	A failure occurred after the patient was admitted to an in-patient service but was still physically in the ED	N/A
**Practitioner-based errors**		
Major cognitive error	An error in cognition which represents serious mismanagement in a knowledge area basic to emergency	Failure to diagnose and treat ST-elevation myocardial infarction
Cognitive error	An error in cognition which represents mismanagement which is either less serious than a major cognitive error or in an area less basic to emergency medicine	Failure to consider the institutional antibiogram during antibiotic selection for treatment
Missed radiographic finding	An error in interpretation of a radiographic study that did not reach the level of a cognitive or major cognitive error	Missed fracture on radiographic interpretation that was still splinted based on clinical suspicion
Policy deviation	An error in following a clinical or administrative policy, guideline or standard practice that does not reach the level of cognitive or major cognitive error	Failure to alert the transplant service when a transplant patient is in the ED
Procedural error	A technical error during performance of a procedure that does not reach the level of a cognitive or major cognitive error	Insufficient sterile technique

All incident reports received by the ED during the study period were recorded prospectively in a secure, quality improvement database. The data included the respective source of each incident report and the results of the peer review analysis. The entire peer review process, including the database and practitioner written responses, was structured so that it complied with state peer review legal discoverability protection requirements.

Sources of incident reports were assigned to one of two broad source group categories: those that were generated by health care providers (HCPs) and those that were from non-health care providers (non-HCPs). The HCP group was sub-divided into four groups: Self (ED provider self-referral), ED (ED provider), HP (Hospital provider) and OP (Other provider outside the hospital), and the non-HCP group was subdivided into three groups: PFM (Patient and family members), Admin (Hospital administration), and Risk (Risk management) for a total of seven sub-groups (Table 2). In the case that reports were submitted by multiple sources for one case, the first report received by ED leadership was identified as the source.

**Table 2:** **Sources of incident reporting**

**Source**	**Definition**
Self	An ED practitioner who was directly involved in the care of the patient when the perceived patient safety incident occurred
ED	An ED practitioner not directly involved in the care of the patient at the time that the perceived patient safety incident occurred
HP	A practitioner within the hospital that is not an ED practitioner or a clinical committee within the hospital (for example - the Stroke Care Committee)
OP	A health care practitioner from outside the hospital
PFM	A patient or family member
Admin	Central hospital management forwarding a concern from an outside agency (for example - an insurance company generated concern)
Risk	Risk management; a hospital administrative unit responsible for risk assessment and quality management.

For the present study, a retrospective analysis of the existing peer review database was performed to determine the PSI capture rate of incident reports from each of the groups and sub-groups. Specifically, each incident report in the database was reviewed to determine the source group and sub-group as well as if any failures or errors were identified for that case indicating that a PSI had occurred.

### Outcomes and analysis

The primary outcome of this study was the frequency of PSI capture based on the source of the incident report. Frequency of PSI capture was expressed as a percent. A two-tailed Fisher’s exact test was used to determine if the capture rates differed between the HCP and non-HCP groups. We similarly compared the frequency of PSI capture within the sub-groups.

## Results

Over a period of 18 months, 353 incident reports were submitted to the ED, and 108 (31%) resulted in the discovery of one or more PSIs. Of the 353 reports, 351 were classified into a source group (Table 2). The reporting source was not recorded in two cases in the database, so they were classified as “unknown”. The frequency of reports for each source group is summarized in Table 3. HCPs submitted 211 reports of which 94 led to PSI capture (44.5%), and non-HCPs submitted 126 reports of which 14 led to PSI capture (10.0%). HCP-generated reports resulted in PSI capture more frequently than those generated by non-HCPs (p < 0.0001).

**Table 3:** **Incident reports and patient safety incident capture by source group**

**Source group**	**Number of reports**	**Percent of total reports**	**Number resulting in PSI identification**	**PSI capture rate**	**p-value**
Healthcare Providers (HCP) n = 211					
Self	40	11.3%	18	45.0% (18/40)	p = 0.99
ED	69	19.5%	31	44.9% (31/69)	
HP	90	25.5%	40	44.4% (40/90)	
OP	12	3.4%	5	41.7% (5/12)	
Non-Healthcare Providers (Non-HCP) n = 140					
PFM	112	31.7%	9	8.0% (9/112)	p = 0.14
Admin	4	1.1%	0	0.0% (0/0)	
Risk	24	6.8%	5	20.8% (5/24)	
**Other**					
Unknown	2	0.6%	0	0.0% (0/2)	n/a
**Overall Total**	353	100%	108	30.6% (108/353)	n/a

Among the subcategories of HCPs and non-HCPs, PSI capture ranged from 41.7-45.0% and 0–20.0% respectively. Reports submitted by the four different HCP subcategories and by the three different non-HCP subcategories did not demonstrate within-group differences with respect to the frequency of PSI capture (p = 0.99 and p = 0.14 respectively) (Table 3).

## Discussion

The results of this investigation of ED incident reports revealed two key findings. First, incident reports submitted by HCPs led to the capture of PSIs nearly 4.5 times more frequently than those submitted by non-HCPs. And second, while HCP-generated incident reports were more likely to result in PSI capture, they resulted in capture only less than half the time.

Given that HCPs are likely to have more insight into healthcare delivery systems than non-HCPs, the finding that HCP-generated reports led more frequently to PSI discovery may not be altogether surprising. More remarkable however was the finding of over a four-fold difference in resultant PSI discovery between these groups. The non-HCP category in our sample was dominated by reports submitted by patients and families. Data from previous investigations have suggested that patient and family concerns often are related to incomplete or delayed relief in symptoms, suboptimal practitioner communication or billing related to their medical services [4,5,9]. The design of this study did not address this topic directly as the specific concerns prompting the incident reports were not systematically recorded in the database, however our experience was consistent with these previous investigations. Of note, while concerns generated by patients and families most often did not lead to PSI identification, we feel strongly that their feedback is still highly valuable for understanding and improving the patient experience.

With regards to this investigation’s second key finding of HCP-generated incident reports capturing PSIs 44.5% of the time, the reasons for this seemingly low capture rate are unclear. Reports from each HCP sub-group resulted in PSI identification at a similar frequency regardless of the HCP’s role or if they were directly involved in the care in question. For providers not directly involved in the care in question (ED, HP and OP), the relatively low percentage of capture could be due to the fact that they were not present during the care in question and therefore were biased toward presuming that an adverse outcome was due to a preventable failure or error. However, this does not explain the capture rate for self-reporting. Perhaps self-reporters were biased by emotion or a sense of personal responsibility when adverse outcomes occurred. In any case, the observed over-reporting by all types of HCPs can be interpreted as a promising sign of their engagement in reducing PSIs. The observed over-reporting by HCPs in this study may appear to contradict previous studies suggesting that HCP under-reporting is a common barrier to improving patient safety [3,9], however the two phenomena are not necessarily mutually exclusive. Based on the prior literature and the results of this study, it is likely that HCP under-reporting and over-reporting are occurring simultaneously. The implication is that HCPs are engaged in patient safety improvement, but there is opportunity to better educate them about patient safety. Optimizing practitioner education in this area may improve over and under-reporting simultaneously. Ultimately reaching a capture rate of 100% may be unrealistic. Furthermore, if a perfect capture rate is attained, it is likely that incidents are going under-reported. To our knowledge, this is the first study reporting capture rates from incident reports, so there are no other data to assist with benchmarking. While we cannot definitively conclude that the rate observed in this study is low, we believe that a capture rate of 44.5% from HCP incident reports can be improved. It stands to reason that the ideal rate may be between the observed rate and 100%. In our institution, we are considering an initial goal of 75% as we continue to improve our incident reporting processes.

While education related to patient safety may be a viable mechanism to improve the PSI capture rate for HCP incident reports, the mechanism to improve them for non-HCP reports is less clear. A prior study reported utilizing structured patient interviews to elicit ED patient feedback rather than self-generated concerns, however the capture rate was still low (9%) [10]. Despite the low rate similar to that observed in our study, we believe that structured patient interviews may still be a promising option. To improve the capture rate of patient and family incident reporting, perhaps the structured interview tool previously reported requires redesign or perhaps follow-up interviews should be directed only toward patients that are high risk for having been subject to a PSI.

Incident reporting by HCPs as well as patients and family members traditionally has been cited as an important tool for PSI identification [9,11-14]. Recently however, some research has shown that while incident reporting can detect latent errors, it may miss a large fraction of PSIs [15]. More proactive and systematic surveillance techniques have been suggested as superior for improving patient safety [15-18] Systematic surveillance techniques however may have potential shortcomings including the fact that they may miss PSIs that do not fit strict systematic surveillance criteria [15,16]. For this reason, we believe that incident reporting and systematic surveillance complement each other, and there is in fact a role for both in healthcare quality improvement [19]. Therefore, gaining a better understanding of incident reporting is important so that we may continue to improve the effectiveness of this incident identification modality.

Previously we have published results of an investigation of the type, frequency, and resultant harm related to PSIs that were discovered via the ED-based incident reporting system [20]. The purpose of the present study was not to review these details again but to investigate the utility of incident reports from different sources in leading to PSI discovery in general. Nevertheless, it is worth noting that in the prior publication, we sought to broadly classify PSIs into those related to either practitioner-based error or systems failures. We are aware that this approach differs from the more detailed categorization scheme used by the National Health Service in the United Kingdom well as that which has been proposed by the World Health Organization [21,22]. Therefore, directly comparing our findings of PSI types with either of those systems would be difficult.

This study of incident reports has potential limitations that warrant consideration when interpreting the results. First, the peer review process was designed to be systematic and as objective as possible; however any review process of incident reports necessarily must involve human interpretation. Barriers for impartial decision making among peers are known to exist, and hindsight bias is likely unavoidable in determining medical errors [23]. A range of measures were designed into the review process to mitigate the influence of biases (discussed in the Methods section), but having completely removed all bias was unlikely. A second potential limitation of the study was the fact that the peer review database only included the original source when more than one report for a case was received. In our anecdotal experience however, very few cases involved reports from both HCPs and non-HCPs, and there was no apparent trend in whether HCPs or non-HCPs were the first to report. The reason for this may have been that non-HCPs also had easily accessible mechanisms to submit incident reports in our institution. In addition to other mechanisms, non-HCPs could submit concerns verbally to the “Patient Care Services” department at any time including at the time of service. Another potential limitation of this study was that it was performed at a single center and focused on EM as opposed to multiple medical specialties. With regard to the generalizability of our study within EM, the frequency of incident reports we observed (0.36% of ED patient encounters) is consistent with other published ED studies which cite a range of 0.012% to 0.61% in EDs with 13,000 to 94,000 patient encounters per year [24,25]. While it cannot be determined if the distribution of incident reporter type is consistent with other EDs, the overall frequency of incident reporting is similar to other reports in the literature. The applicability of this study beyond EM is less clear. It may be the case that incident reporting is more likely to occur in EDs because nearly all care is handed off to other HCPs who naturally review the care that has been delivered prior to their involvement. However the fact that incident reports from non-ED HCPs resulted in similar PSI capture rates as those from ED HCPs in the study provides some promising evidence that the results may be generalizable to other specialties.

## Conclusion

Any incident report, regardless of its source, has the potential to lead to PSI discovery and ultimately result in quality improvement. This study revealed that HCP-generated reports led to PSI capture much more frequently than reports from other sources; however they still resulted in capture less than half the time. These two observations together provide promising evidence that there may be opportunity to further improve the already valuable feedback from HCPs and that quality improvement programs may likely benefit by prioritizing strategies to further increase and optimize feedback from this group. By comparison, non-HCP generated concerns appear to be relatively less valuable for PSI identification. Quality improvement leaders might consider implementing programs to solicit directed feedback from patients and families in order to improve the patient safety incident capture rate, however the potential benefit of these efforts remains unclear to date. Further research is warranted to:

1. determine the most effective strategies to further increase and improve HCP incident reporting
2. determine if soliciting directed patient and family feedback may improve PSI capture and
3. determine if patient feedback systems targeted at subgroups of ED patients that are at higher risk of safety incidents may improve PSI capture rates from this group.
